# T Cells Chemotaxis Migration Studies with a Multi-Channel Microfluidic Device

**DOI:** 10.3390/mi13101567

**Published:** 2022-09-21

**Authors:** Yang Liu, Xiaoou Ren, Jiandong Wu, John A. Wilkins, Francis Lin

**Affiliations:** 1 Department of Physics and Astronomy, University of Manitoba, 30A Sifton Rd, 301 Allen Bldg, Winnipeg, MB R3T 2N2, Canada; 2 Institute of Biomedical and Health Engineering, Shenzhen Institute of Advanced Technology, Chinese Academy of Sciences, Shenzhen 518055, China; 3 Manitoba Centre for Proteomics and Systems Biology, University of Manitoba and Health Sciences Centre, 799 JBRC, 715 McDermot Ave, Winnipeg, MB R3E 3P4, Canada

**Keywords:** chemotaxis, T cells, migration, microfluidic device, multi-channel

## Abstract

Immune surveillance is dependent on lymphocyte migration and targeted recruitment. This can involve different modes of cell motility ranging from random walk to highly directional environment-guided migration driven by chemotaxis. This study protocol describes a flow-based microfluidic device to perform quantitative multiplex cell migration assays with the potential to investigate in real time the migratory response of T cells at the population or single-cell level. The device also allows for subsequent in situ fixation and direct fluorescence analysis of the cells in the microchannel.

## 1. Introduction

Lymphocyte circulation is a critical aspect of immune surveillance and activation, facilitating the effective targeting of cells to sites of antigen presentation or inflammation. Lymphocyte migration is controlled in part by the restricted tissue expression of chemokines that selectively recruits subsets of lymphocytes expressing the corresponding chemokine receptors [1,2]. A detailed understanding of the underlying mechanism of these processes is essential for the development of approaches to potentially manipulate immune recruitment in health and disease [3].

A limited number of assay systems have been developed to measure and monitor lymphocyte migration in vitro [3,4]. However, the recent introduction of microfluidic devices offers several unique capabilities [5,6] including the capacity to (1) generate defined, stable gradients [7,8,9]; (2) directly visualize and quantify cell movement in real time [10,11]; and (3) introduce complex multicellular microenvironments [12,13]. These features complement the capacity to perform comparative multiplex assays with limited numbers of potentially restricted cells and reagents. The architecture of the devices with their docking structure is also helpful as it aligns the cells to one side of the channel prior to the induction of migration. This provides the opportunity to visualize multiple cells at similar stages of migration.

This chapter describes methods for the production and use of a multichannel flow-based microfluidic device to quantify and monitor the migration of activated human peripheral blood T cells and T leukemic cell lines. The current microfluidic device offers increased versatility and qualifies as an attractive higher throughput chemotaxis assay for the study of lymphocyte chemotaxis.

## 2. Materials

### 2.1. Design and Fabrication of the Multi-Channel Radial Microfluidic Docking Device (D^9^-Chip)

1. Photomask (FineLine Imaging, Colorado Springs, CO, USA)
2. SU-8 photoresist (Kayaku Advanced Materials, Inc., Westborough, MA, USA)
3. 3-inch silicon wafer (Silicon Materials Inc., Reno, NV, USA)
4. Polydimethylsiloxane (PDMS) (Sylgard 184; Dow Corning, Texas City, TX, USA)
5. Air Gas Duster (Amazon.ca or local retail)
6. Ultraclean glass slides (ThermoFisher, Waltham, MA, USA)

### 2.2. Cell Culture and Reagent Preparation

1. RPMI 1640 culture medium, HEPES, L-Glutamine (catalog# 22400089, ThermoFisher, Waltham, MA, USA)
2. Fetal bovine serum (catalog# 12484-028, GibcoTM, ThermoFisher, Waltham, MA, USA)
3. Penicillin-Streptomycin (5000 U/mL, catalog# 15070-063, GibcoTM, ThermoFisher, Waltham, MA, USA)
4. Bovine serum albumin (catalog# SH30574.02, Hyclone Laboratories, Logan, UT, USA)
5. Phosphate buffered saline (PBS) without Ca^2+^ and Mg^2+^ (catalog# SH30378.03, Hyclone Laboratories, Logan, UT, USA)
6. Anti-human CD3 (catalog# 300302, BioLegend, San Diego, CA, USA)
7. Anti-human CD28 (catalog# 302901, BioLegend, San Diego, CA, USA)
8. Interleukin-2 (IL-2) (US National Cancer Institute, Bethesda, MD, USA)

### 2.3. T Cell Migration Experiment

1. Fibronectin solution (1 mg/mL, catalog# FC010, MilliporeSigma, Burlington, ON, Canada)
2. SDF1alpha (catalog# 300-28A, PeproTech, East Windsor, NJ, USA)
3. FITC-Dextran (10 kD, catalog# FD10S, Sigma-Aldrich, Oakville, ON, Canada)
4. Silicone oil (catalog# A12728-22, Alfa Aesar, Ward Hill, MA, USA)

### 2.4. Actin Staining and Visualization for Cell Migration Experiment

1. Paraformaldehyde Solution, 4% in PBS (catalog# J61984-AK, Alfa Aesar, Ward Hill, MA, USA)
2. Phalloidin-iFluor 594 Reagent (catalog# A12381, ThermoFisher, Waltham, MA, USA)
3. Nikon Confocal Microscope (Nikon Ti-U) with fluorescent imaging capabilities (Nikon Instrument Inc., Melville, NY, USA) and environmental temperature control chamber (InVivo Scientific, Salem, SC, USA)

### 2.5. Data Analysis Software

1. Image J/Fiji (Version 1.53, Wayne Rasband and contributors, NIH, Bethesda, MD, USA)
2. GraphPad Prism 6.0 (Version 6.0, GraphPad Software, Inc., San Diego, CA, USA)

## 3. Procedure and Expected Results

### 3.1. Microfluidic Device Preparation

#### 3.1.1. D^9^-Chip Design

**Figure 1:**
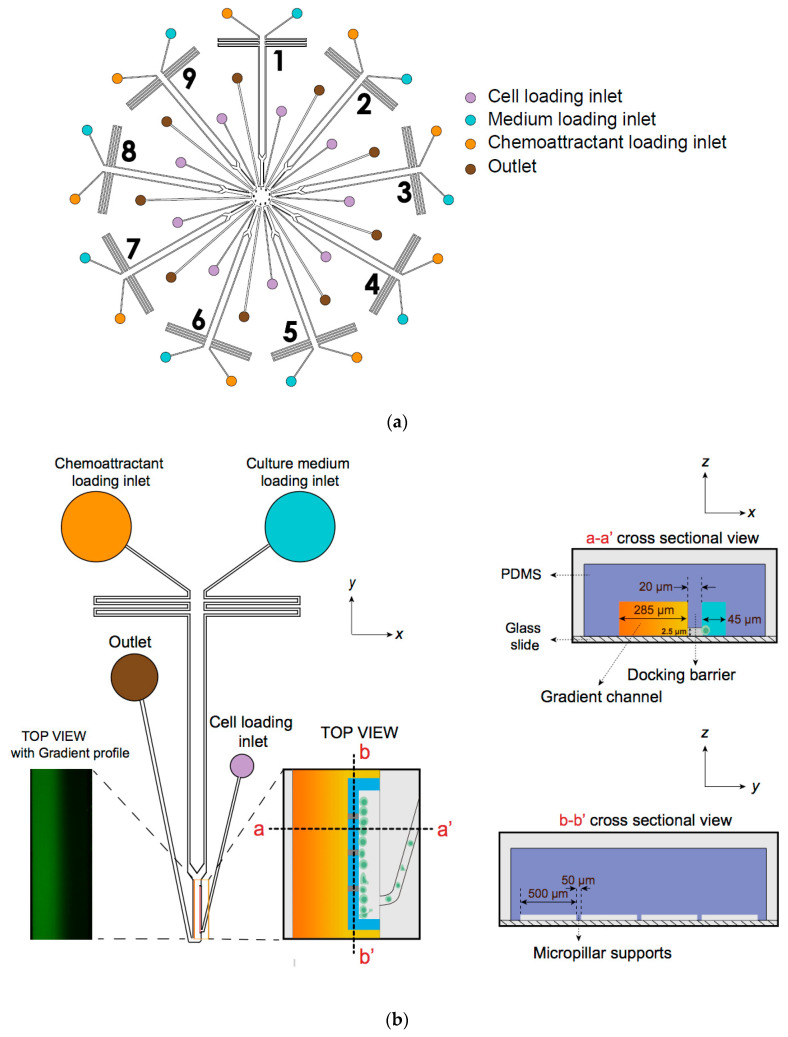
Illustration of the multi-channel radial microfluidic device. (**a**) Design of the nine-unit whole chip and illustration of the inlets and channels in the cells chemotaxis experiment. It provided independently controlled gradients by adding chemoattractant and medium to different loading inlets. (**b**) The cross-section view and top view of a single unit illustrated the cell docking structure and pillar support. The design of the cell docking structure aims to align cells and separate them from the cell loading channel and the main gradient channel.

1. The D9-Chip pattern was designed using AutoCAD 2018 (Version 22.0, Autodesk Inventor, San Rafael, CA, USA) and generated the chip’s geometry.
2. D9-Chip has nine individual units to use, and each unit has a distinct migration channel with a unique cell loading inlet, chemical inlets and outlet (Figure 1).
3. Equilibrated flow from two chemical inlets will generate a stable chemokine gradient by molecular diffusion in pressure driven micro-channels.
4. Three micropillars support the barrier channels from below to increase the structure stability of the channels and prevent collapse during the glass bonding process after plasma treatment.

#### 3.1.2. D^9^-Chip Fabrication

1. The design pattern was printed on a transparent film at high resolution to serve as a photomask.
2. The photomask within the pattern was then projected on a 3-inch silicon wafer (~50 μm high) by UV exposure with SU-8 photoresist.
3. The designed features were fabricated on silicon wafers by two-layer contact photolithography. The first layer defines the cell docking structure and controls the initial position of individual cell in the microfluidic device (Figure 1). The second layer containing the features of the gradient channel was then aligned with the first layer on the same wafer. The specific parameters of the docking structure are referenced in Note 1.
4. After the photolithography, PDMS replica were fabricated using standard soft-lithography technique. Specifically, Sylgard 184 silicone base and curing agent were mixed in a 10:1 ratio by weight to replicate the feature from master mold.
5. The PDMS replica were placed in a petri dish in a vacuum desiccator to remove air bubbles. Alternatively, air bubbles can be blown to the sides of the petri dish using Air Gas Duster.
6. The PDMS replica were baked at 80 °C for 2 h, followed by peeling off from the silicon master mold.
7. One cell loading port (2 mm diameter), two chemical inlets (6 mm diameter) and one outlet (4 mm diameter) were punched out for each unit of the PDMS replica.
8. The channel surface of PDMS replica was cleaned with adhesive scotch tape to remove any residual PDMS fragments.
9. For each device, a glass slide was rinsed in the following order with acetone, isopropanol, DI water and blown dry by airflow in a biosafety cabinet.
10. The PDMS replica and a glass slide were placed inside a Plasma Cleaner Chamber (Harrick Scientific, Pleasantville, NY, USA) and the vacuum was applied for 2–3 min, followed by high power mode of air plasma treatment. This step was used to bond the PDMS replica on the glass slide and restore the hydrophilicity of the PDMS channels for later injection of the solutions.

#### 3.1.3. Substrate Coating

1. The completed microfluidic device was immediately coated with substrate to preserve the hydrophilic ability of the channels.
2. The completed microfluidic devices were directly coated with fibronectin working solution (0.25 mg/mL in PBS) prior to use in cell migration experiments. The high coating concentration was used to achieve a required surface coating density, taking into consideration the low height (~50 μm) of the channels in the microfluidic device [2]. The devices were coated with fibronectin solution changes every 10 min for 1 h in room temperature. The devices were subsequently blocked overnight at 4 °C with 0.4% BSA RPMI 1640 medium.

### 3.2. Chemical Gradient Generation

#### 3.2.1. Principle of Gradient Generation

1. The gradient generation principle in our device is based on the continuous mixing of two laminar flows containing target chemoattractant and medium control solutions in the microchannels [14,15]. The gradient generation in each unit is independent, fast, stable, and well-controlled in a pump-free manner.
2. The pressure difference of the solutions between the two filled chemical inlets and the empty outlet in each unit drives the two flows from the inlets to the downstream main gradient channel and the corresponding outlet.
3. A previously described silicone oil-based pressure-balancing strategy is applied to cover the two chemical inlets using the oil after loading the corresponding solutions [16]. The strategy is based on the immiscibility between the silicone oil and the solutions and to eliminate the pressure difference between the two inlets (Figure 1b).
4. The versatility of the D^9^-Chip design device is in generating single or overlapped gradients by using ad hoc designed microchannels in which adjacent fluid streams contain the chemicals of interest and the medium.

#### 3.2.2. Measurement of Concentration Chemical Gradient

1. The chemical concentration gradient profile was simulated by COMSOL Multiphysics^®^ 5.6 (COMSOL, Inc. Burlington, MA, USA) using laminar flow and chemical diffusion theory and characterized and validated by experimental assay.
2. FITC-dextran was dissolved in DMSO at a stock concentration of 30 mg/mL, which was used to characterize the proper gradient generation by adding a final concentration of 0.6 mg/mL in the chemoattractant solution.
3. FITC-dextran containing chemotaxis solution or complete media control (50–100 μL, depending on the thickness of the device) were loaded into separate chemical inlets on a single channel (Figure 1b).
4. The above solutions were overlayed with silicone oil (20–30 μL, depending on the thickness of the device) to cover the solutions in the two inlets.
5. The fluorescence emitted from FITC-dextran was measured to monitor the gradient profile during the chemotaxis experiment.

### 3.3. Cell Preparation

#### 3.3.1. Preparation of Activated Human Peripheral Blood T Cells (ahPBTs)

1. All human blood-related experiments were carried out in accordance with relevant guidelines and regulations set at the University of Manitoba and The Tri-Council Policy Statement of Ethical Conduct for Research Involving Humans (TCPS). An ethics protocol (Protocol number: HS24865) was approved by the Joint-Faculty Research Ethics Board at the University of Manitoba for collecting blood samples.
2. Human peripheral blood samples were obtained from healthy donors by the recruiting staff employed at the Victoria General Hospital in Winnipeg following the approved protocol procedures. Informed written consent forms were obtained from all participants.
3. Peripheral blood mononuclear cells (PBMCs) were isolated from the blood samples [16,17] and cultured (0.5–1 million cells/mL) in the presence of 1 µg/mL each of anti-human CD3 and anti-human CD28 antibodies in a complete RPMI culture medium (RPMI-1640 with 50 U/mL final concentration penicillin-streptomycin and 10% FBS) for 48 h (37 °C, 5% CO_2_).
4. The activated ahPBTs were subsequently expanded in a complete RPMI supplemented with 12.5 ng/mL of IL-2 for at least 3 days prior to use in migration experiments [14,18,19].

#### 3.3.2. Preparation of Jurkat Cells

1. Jurkat Clone E6-1 (Jurkat, ATCC number TIB-152) is a human T lymphoblastoid cell line derived from an acute T cell leukemia patient [20]. Cells were cultured in RPMI 1640 supplemented with 25 mM HEPES and 2.05 mM L-glutamine, with 10% FBS and Penicillin-Streptomycin (50 U/mL final concentration), and incubated in 5% CO_2_ at 37 °C.
2. Cultures were initiated at a cell density of 10^5^/mL and subcultured when they reached ~10^6^/mL.

### 3.4. Experimental Preparation

#### 3.4.1. Cell Loading

1. Prior to use in migration assays, the cells were collected and incubated in serum and a BSA-free RPMI 1640 medium at a concentration of 5–10 × 10^5^/mL for 3–4 h. This step reduced the levels of spontaneous migration in the cells.
2. The cells were collected and resuspended in a RPMI 1640 medium containing 0.4% BSA. The cells were adjusted at a concentration of 5–10 × 10^6^/mL before gently loading 5–10 × 10^4^ cells in a 10–15 μL RPMI 1640 medium containing 0.4% BSA to the cell loading port in each unit of the fibronectin-precoated radial microfluidic device.
3. Cell suspension was loaded into each cell loading port. The cells accumulated at ~2.5 µm height docking barrier of the device as previous described [16,21].
4. Once approximately 1000–2000 cells were allowed to align next to the gradient channel, the migration analysis was initiated. This alignment of the cells at a common “start” position offered optimal conditions for accurate analysis of migration.

#### 3.4.2. Chemoattractant Preparation and Loading

1. Each experiment has an SDF-1α and a control (RPMI 1640 medium containing 0.4% BSA) channel.
2. SDF-1α (100 nM) in a RPMI 1640 medium containing 0.4% BSA with 0.006% (*w*/*v*) FITC-dextran (10 kD) is prepared from stock for each experiment.
3. The chemoattractant solution and cell migration medium control were loaded into the corresponding inlets. Specifically, the inlet which is closer to the cell loading port was filled with a migration medium control (RPMI 1640 medium plus 0.4% BSA) and the other inlet was filled with the test solution (i.e., chemoattractant).
4. Upon loading the corresponding solution, the inlets were covered and connected with silicone oil (density = 0.963 g/mL) to balance the pressure difference between two inlets. In addition, the oil coverage strategy reduces the solution evaporation from the inlet wells; it can also help maintain a pressure difference to drive the solution from the inlets to outlet.

#### 3.4.3. Imaging System and Data Collection

**Figure 2:**
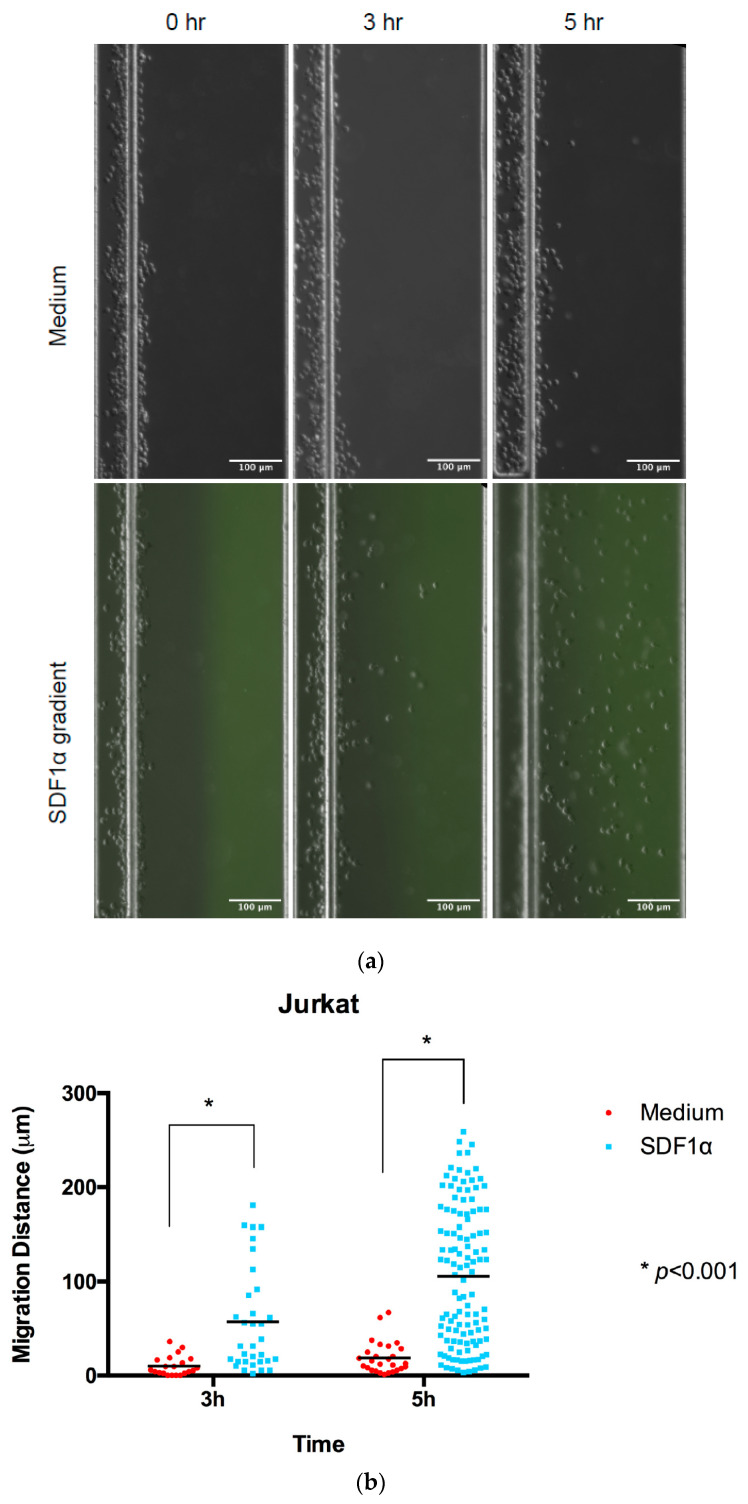
Chemotaxis of Jurkat cells in multi-channel radial microfluidic device. (**a**) Representative Jurkat cells distribution images in the channel at the beginning and the 3 h and 5 h chemotaxis experiment in a 100 nM SDF-1α gradient. (**b**) Quantitative migration distance analysis for the experiments in (**a**). The results are presented as the average value ± standard error of the mean (SEM). * indicates *p* < 0.001.

**Figure 3:**
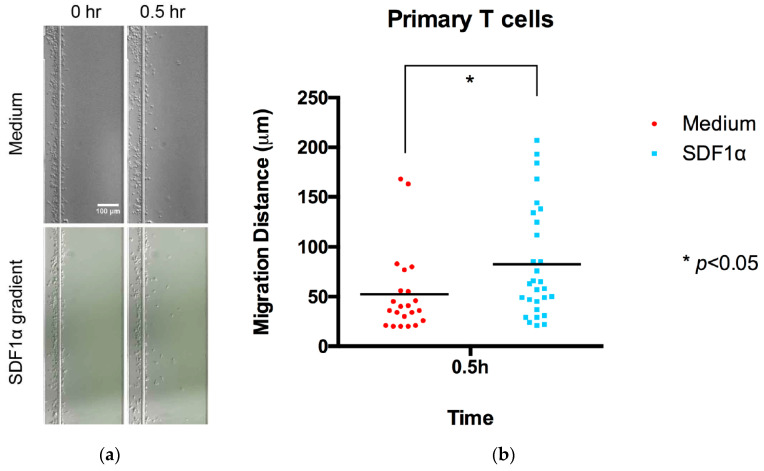
Chemotaxis of primary T cells in the radial microfluidic device. (**a**) Representative primary T cells distribution images in the channel at the beginning and the 0.5 h chemotaxis experiment in a 100 nM SDF-1α gradient. (**b**) Quantitative migration distance analysis for the experiments in (**a**). The results are presented as the average value ± standard error of the mean (SEM). * indicates *p* < 0.05.

1. The loaded microfluidic devices were placed in an environmental chamber mounted on a Nikon Confocal Microscope (Nikon Ti-U) with fluorescent imaging capabilities (Nikon Instrument Inc., Melville, NY, USA) with the appropriate filter at Ex/Em = 490/520 nm and a capacity to maintain a temperature at 37 °C and 5% CO_2_ atmosphere during the imaging process.
2. Jurkat cell migration was monitored using images taken at 0, 3 h, and 5 h time points to calculate the displacement of the cells by comparing the positions at different time points with Image J (Figure 2). Comparisons of individual cell displacement in the chemotaxis (SDF-1α) or medium control group were plotted by measuring the distance cells migrated from their original position.
3. Primary T cells migration was monitored using images taken at 0 and 0.5 h, as these cells displayed a higher migration rate (Figure 3). In addition, the cell migration pattern of primary T cells within the channel was real-time monitored under microscopy (Video S1). The entire videos are for 30 min experimental periods, and the video playback speed is 10×.

#### 3.4.4. In Situ Cell Labelling

**Figure 4:**
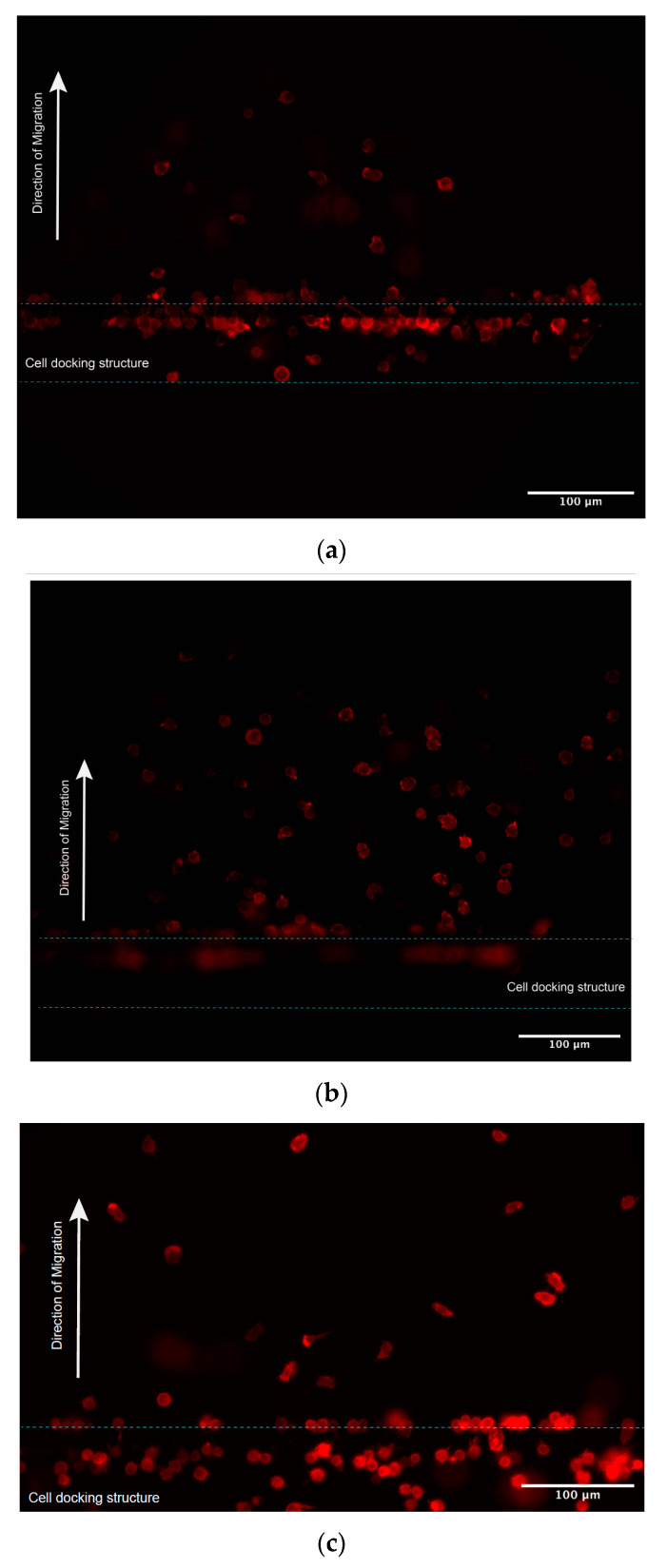
Cells were stained by phalloidin to observe actin filament expansion. (**a**,**b**) Jurkat cells were fixed at the end of 5 h chemotaxis migration experiments, followed by F-actin staining with Phalloidin-iFluor 594 reagents working solution. (**a**,**b**) Representive different cells locations under different focal planes. (**c**) Primary T cells were fixed at the end of 0.5 h chemotaxis migration experiments, followed by F-actin staining with Phalloidin-iFluor 594 reagents working solution.

**Figure 5:**
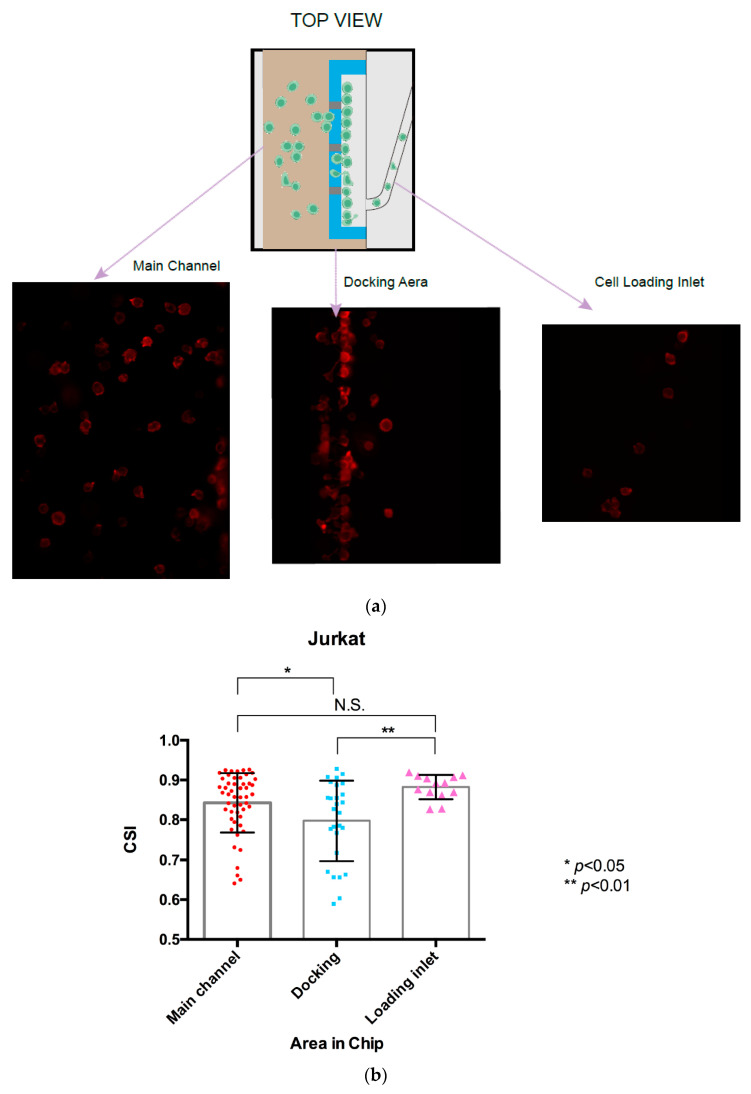
Analysis of the Jurkat cell shape index (CSI) in the chemotaxis migration assay. (**a**) Illustration of the actin cytoskeleton staining of Jurkat cells located in the different areas of the microfluidic device, starting from the cell loading inlet, the docking area and the gradient channel. (**b**) Calculation CSI of the Jurkat cells located in the different area of the chip. The results are presented as the average value ± standard error of the mean (SEM). * indicates *p* < 0.05. ** indicates *p* < 0.01, N.S. indicates not significant.

1. Migrating cells were also fixed and labelled in situ for detailed morphological analysis.
2. The chemoattractant solution and medium were removed by gently pipetting from the inlet and outlet wells. It is important that the interior compartments do not dry out during this process. The channels were rinsed with 90–100 µL of PBS to inlet and outlet wells for 5 min at room temperature. After rinsing with PBS, the cells were fixed 30 min at room temperature by adding 90–100 µL of 4% PFA to inlet and outlet wells in order to completely fill the channel with fixation solution.
3. The 4% PFA solution was removed from the inlet and outlet wells in the chip, the channels were rinsed with 90–100 µL of PBS to inlet and outlet wells for 5 min at room temperature, taking care not to introduce air bubbles. This was repeated twice.
4. Fresh phalloidin staining solution (1:1000 dilution of stock solution) was prepared in PBS/1% BSA and incubated for 30 min at room temperature in the dark.
5. The solution was removed from the wells in the chip, the cells were rinsed with PBS (3 times/5 min each) to remove excess phalloidin conjugate.
6. The final wash was removed and 50–60 µL PBS was added in the device to prevent the channel from drying.
7. The cells were then observed and photographed using a Nikon Confocal Microscope (Nikon Ti-U) fitted with an appropriate filter at Ex/Em = 590/618 nm (Figure 4).
8. The cell shape index (CSI) was calculated through the cell area and perimeter measurements for the cells at different locations in the gradient channel. Figure 5a shows successive snapshots of the cell morphology changes during the chemotactic migration process with the phalloidin-stained actin filaments, starting from the cell loading inlet through the docking area to the main migration channel. The CSI was defined for the cell morphology analysis during the chemotaxis migration process. Comparing the CSI between different cell locations in Figure 5b, cells appeared to stretch and elongate while crossing the 2.5 μm thickness barrier area revealing the quantitative cell stretching and spreading information. Cell shape changed from the cell loading inlet to the docking area with CSI ranging from 0.88 in the circular-shaped cells to 0.80 in the elongated cells; from the docking area to the main gradient channel, the CSI ranged from 0.80 in the elongated cells to 0.84 in the circular-shaped cells.

#### 3.4.5. Statistical Analysis

1. Student’s *t*-test was used to compare the different groups using Prism; *p* < 0.05 was considered statistically significant.
2. The cell area and perimeter were determined using Image J. CSI was calculated from the following formula [22]: A is area of the cell, P is perimeter.

$$\mathrm{CSI}=\frac{4\pi A}{P^{2}}$$

## 4. Conclusions

The nine-unit radial microfluidic chip provides a platform that permits the study of cell migration and chemotaxis at a higher throughput and may serve as an attractive discovery tool to quantify the influence of compounds that influence the chemotaxis of inflammatory cells.

## 5. Notes

### 5.1. Cell Docking Structure in a Radial Microfluidic Device

The cell docking structure used in this device is specifically tailored for the cell type under investigation. In the case of T cells or neutrophils, a docking device thickness of ~2.5 µm was used. However, for most types of cancer cells, such as the breast cancer cell line MDA-MB-231, a ~7 µm barrier is used [16]. The guiding principle for determining the thickness of the docking area barrier is to exclude random walk migration by designing the height slightly lower than the cell diameter which still permits and selects for deliberate cell migration into the gradient channel. The depth of the second layer of the device is around 44–46 µm.

### 5.2. Fibronectin Coating Treatment on the Device for a Better T Cell Migration Environment

Fibronectin concentration is vital to affect the migratory characteristics of the Jurkat cells. CD49e/CD29 (α5β1, VLA-5), which recognizes the RGD-containing fibronectin binding site, and CD49d/CD29 (α4β1, VLA-4), which binds the CS-1 region on fibronectin [23], control the cells’ attachment and migration movements according to the leukocyte activation status [24,25,26]. In this case, we applied a relatively high concentration of fibronectin coating in microchannels for 1 h, and repeated the flash every 10 min with fresh fibronectin solution to achieve the required surface coating density in the channels.

### 5.3. FITC-Dextran Fluorescence Additive in the Chemotaxis Gradients

FITC-dextran can be added to the SDF-1α containing solution as a direct indicator of the gradient generation in the microchannel. In such cases, care should be taken to ensure that the FITC-dextran does not interfere with the chemoattractant molecules. A FITC-dextran conjugate with a molecular weight similar to the chemoattractant molecules should be used. Most chemokines have molecular weights ranging from 8 kD to 10 kD (e.g., SDF1α [27], IL-8 [28,29]).

### 5.4. Actin-Based Protrusions of Migrating Cells

Phalloidin staining of the actin cytoskeleton has been considered as the most reliable method of accurately labelling F-actin in fixed migration cell. In this case, we applied fluorescence iFluor 594-conjugated phalloidin to visualize the cellular F-actin intensity by specific binding. In further morphology analysis, we calculated the cell shape index CSI to estimate the chemotaxis-induced shape transition of the cells during the migration process, especially by using this docking structure microfluidic device. In this case, we estimated the cells must remodel their morphology through the cytoskeleton to be more elongated in the docking area, preparing them to migrate through the thin barrier channel in response to the chemoattractant gradient. However, morphological cell elongation is significantly reduced in the gradient channel compared to the docking area but also higher than cells in the loading inlet not exposed to chemoattractant, although not significantly in the current CSI analysis.

The hypothesis of actin-based protrusions of migrating cells and migration of cells powered by actin could be further demonstrated in 3D matrix chips [30].

## Data Availability

The data presented in this study are available in the article.

## Supplementary Materials

The following supporting information can be downloaded at: https://www.mdpi.com/article/10.3390/mi13101567/s1, Video S1: Real-time cell migration pattern of primary T cells under microscopy.
